# Tissue fluidity: biophysical shape-shifting for regeneration

**DOI:** 10.1038/s41392-024-02068-9

**Published:** 2024-11-26

**Authors:** Zitong C, Zhenyu C, Y. Rinkevich

**Affiliations:** 1 Institute of Regenerative Biology and Medicine, Chinese Institutes for Medical Research, Fangtai District, Beijing, 100069 China; 2 Capital Medical University, Fengtai District, Beijing, 100054 China

**Keywords:** Self-renewal, Skin stem cells

**Fig. 1:**
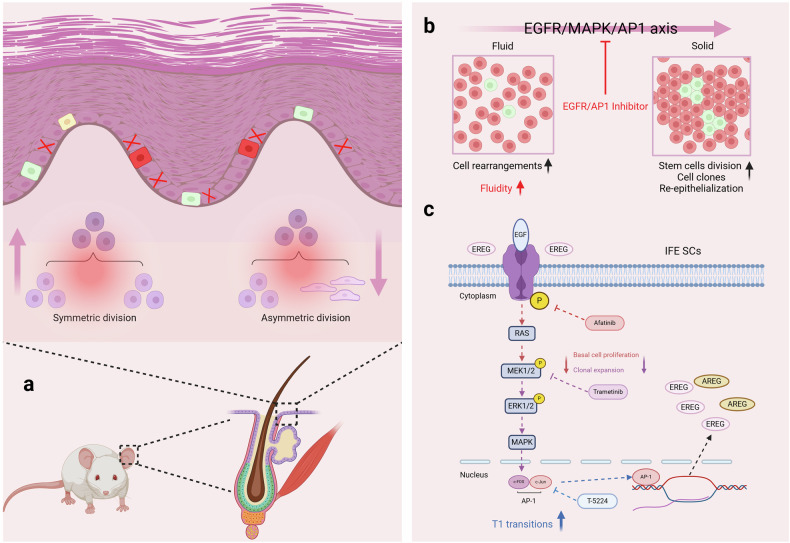
A graphical summary of Sarate et al.‘s findings and the existing mechanisms of skin tissue fluidity. **a** The inducible basal cell ablation can result in up-regulated symmetric division. **b** The EGFR/MAPK/AP1 axis showed its function of regulating the fluid-solid state transformation. **c** The detailed EGFR/MAPK/AP1 axis and potential mechanism of EGFR/AP1 inhibitor

In a recent study published in *Cell*, Sarate et al. developed a novel mouse epidermal injury model through genetic ablation of basal epidermal cells, revealing a physical transition of epidermal cells from solid to fluid-like states. Through combined omics approaches, they discovered that tissue fluidization is regulated by the EGFR/MEK/AP1 signaling axis, opening new opportunities to reactivate stem cells for wound repair^1^ (Fig. 1).

From embryonic development to adult injury, epithelial cells can transition from a solid-like into a fluid-like biophysical state to support cellular activation and tissue repair. Fluid-like states are visualized across tissue scales as changes in the position of individual cells or multicellular collectives relative to one another. Biophysical transitions of cells in tissues, from solid-like to fluid-like states, occur, for example, during unjamming and intercalation between cells, during increased cellular density, and also manifest in response to extracellular matrix such as basement membrane remodeling.^2^ All of these may reflect dynamic rearrangements of cells and potentially of the extracellular matrix surrounding cells to support the fluid-like movements of cells and, ultimately, tissue generation (in embryogenesis) or regeneration.

The skin represents a valuable model for exploring the cellular dynamics during injury repair. The outer epidermal layer of the skin contains pilosebaceous units, including hair follicles and sebaceous glands linked to the interfollicular epidermis via the infundibulum. The innermost basal epidermal cells are proliferative, and in the healthy state, they differentiate asymmetrically into different types of epidermal cells as they move upward to contribute to the interfollicular, hair follicle, and infundibulum.^1^

A recent study in this issue of *Cell* by Sarate et al. tracks the biophysical nature of basal epidermal cells in a mouse injury model where basal epidermal cells are depleted, uncovering novel mechanisms that are associated with tissue fluidization and regeneration driven by EGFR/AP1 in the epidermis. The authors developed a novel injury model whereby basal cells in the interfollicular epidermis are ablated by diphtheria toxin in a doxycycline-dependent manner.^1^ Basal cell lineage ablation leads to the activation and recruitment of neighboring and more distant basal cells to regenerate the epidermis. Live imaging of individual clones in living mice revealed changes in the biophysical states of cells consistent with fluid-like behaviors when more than half of the basal cells are lost (Fig. 1a, b). Using their genetic injury model, Sarate et al. used chromatin accessibility and transcriptome sequencing to determine the transcriptional and epigenetic landscape associated with fluidization. They found increased accessibility of AP1 in regenerative conditions. As AP1 binds to Jun and Fos transcription factors, single-cell RNA-sequencing was performed, indicating Jun-Fos regulation is upregulated in the regenerative cells during fluidization states.

Further, tissue fluidization correlated with the upregulation and phosphorylation of EGFR. EGFR activation links to multiple downstream transcription effectors of cell growth, proliferation, and survival. Notably, the authors used a small-molecule inhibitor to define the functional roles of the EGFR/MAPK axis in tissue fluidization during basal cell ablation by affecting cell-cell adhesion and actomyosin-driven cortical contractility. Indeed, from this injury model, tissue fluidity could be accelerated by applying EGFR/AP1 inhibitors in vivo, suggesting that EGFR/AP1 inhibitors could stimulate tissue fluidity during the injury state.

A significant strength of this study was combining live imaging with mathematical Voronoi modeling that integrates cell position and topology parameters after basal-cell ablation. These indicated two topological transitions during epidermal regeneration: proliferative-driven and non-proliferative-driven fluidization events.

Phase transitions in tissue states from a rigid to a flowing state or vice versa define much of what happens in many biological processes, especially during early development and diseases such as cancer. For example, dynamic changes in the biophysical properties of cancer cells occur during cancer progression, and fluidization predicts tumor aggressiveness in vivo.^3^ During development, measuring cell flows in the growing zebrafish tailbud^4^ shows a transition in tissue fluidity that enables a directed posterior flow of composite tissue and is affected by inhibition of Wnt or Fgf signaling or cadherin, two functions. Another example comes from studying avian skin development.^5^ Yang and colleagues identified material property changes (e.g., elasticity) from solid to fluid-like. Specifically, fibroblast growth factor (FGF) “solidifies” a dermal analog needed for hair follicle budding and formation, whereas bone morphogenetic protein (BMP) maintains fluidity in its margin. Thus, a morphogenic core is generated through separate biophysical transitions.

Future experiments that explore the links between cell and microenvironment composition dynamics in controlling tissue fluidization could provide novel translational opportunities to coax stem cell activation and tissue regeneration.
